# Supplementary material for: Aberrant Effective Connectivity Within and Between the Default Mode, Executive Control, and Salience Networks in Chronic Insomnia Disorder—Toward Identifying the Hyperarousal State
Source: Biomedicines. 2025 May 24;13(6):1293. doi: 10.3390/biomedicines13061293 (PMC12189937; doi:10.3390/biomedicines13061293)
Supplement: Supplementary file 1 [file biomedicines-13-01293-s001.zip › biomedicines-3647155-supplementary.pdf]

Supplementary Table S1: Regions of interest (ROI) with their Montreal Neurological Institute (MNI) coordinates for Model 1

ROI	X	Y	Z	Brodmann area
Medial prefrontal cortex	3	54	-2	14,11
Ventral Medial prefrontal cortex	44	52	-2	25
Dorsal Medial prefrontal cortex	4	30	46	32
Left inferior parietal lobe	-50	-63	32	39,40
Precuneus	-10	-64	24	7
Posterior cingulate cortex	0	-52	26	23,31
Hippocampal formation Left	-24	-11	-18	28
Hippocampal formation Right	24	-12	-20	28

Supplementary Table S2: Regions of interest (ROI) with their Montreal Neurological Institute (MNI) coordinates for Model 2

ROI	X	Y	Z	Brodmann area
Medial prefrontal cortex	3	54	-2	14,11
Precuneus	-10	-64	24	7
Posterior cingulate cortex	0	-52	26	23,31
Hippocamp Left	-24	-11	-18	28
Hippocamp Right	24	-12	-20	28
Anterior insula Left	-34	22	4	13
Anterior insula Right	38	22	3	13
Left dorsal anterior cingulate cortex	-5	14	34	24

Supplementary Table S3: Regions of interest (ROI) with their Montreal Neurological Institute (MNI) coordinates for Model 3

ROI	X	Y	Z	Brodmann area
Medial prefrontal cortex	3	54	-2	14,11
Posterior cingulate cortex	0	-52	26	23,31
Hippocampus Left	-24	-11	-18	28
Hippocampus Right	24	-12	-20	28
Dorsolateral prefrontal cortex	-37	27	44	46
Cingulate gyrus	0	26	31	24,32

One-sample Kolmogorov-Smirnov test results and between-group comparisons of Model 1.

The first model exploring the effective connectivity within the DMN demonstrated that in the healthy control group there were 9 connections that statistically differed from zero namely: the self-inhibitory connections of the MPFC, DMPFC, and PCC, MPFC → IPLL, IPLL → PCC, MPFC → HippocampL, DMPFC → HippocampL, IPLL → HippocampL, and VMPFC → HippocampR. Concurrently, in the patient group the significant connections were: HippocampL → MPFC, DMPFC → VMPFC, MPFC → Precuneus, IPLL → Precuneus, and the self-inhibitory connection of the HippocampL (Suppl. Table S4).

The between-group comparison yielded statistically significant differences in the connectivity strengths of the DMPFC → VMPFC, PCC → IPLL, PCC → Precuneus , MPFC → PCC, and HippocampL → HippocampR connections (Suppl. Table S4).

Supplementary Table S4: Group-wise and between-group connectivity strength analysis in chronic insomnia patients and healthy controls, according to Model 1.

**Connection**	**CID Group**			**HC Group**			**Between group comparison**
	**Mean**	**(±SD)**	**D**	**Mean**	**(±SD)**	**D**	**U**
MPFC - MPFC	-0.24221903	0.330	.200	-0.31748156	0.283	.036*	0.352
VMPFC - MPFC	-0.01536381	0.231	.200	-0.03077512	0.350	.200	0.954
DMPFC - MPFC	0.00489877	0.302	.200	-0.05415228	0.429	.200	0.711
IPLL - MPFC	-0.01093535	-0.341	.200	0.07173248	0.438	.200	0.592
Precuneus - MPFC	0.18197284	0.341	.200	0.11500980	0.557	.200	0.967
PCC - MPFC	0.21433581	0.337	.200	0.17141548	0.389	.200	0.686
HippocampL - MPFC	-0.07388497	0.589	.040*	0.05801032	0.487	.200	0.443
HippocampR - MPFC	-0.28549729	0.502	.149	-0.29787916	0.467	.200	0.639
MPFC - VMPFC	0.06277142	0.212	.148	-0.01209948	0.194	.200	0.104
VMPFC - VMPFC	-0.16904745	0.283	.200	-0.14226924	0.213	.200	0.798
DMPFC - VMPFC	0.20608003	0.342	.018*	0.35445896	0.369	.124	0.049*
IPLL - VMPFC	0.01571581	0.218	.129	-0.01537456	0.201	.200	0.811
Precuneus - VMPFC	-0.02888077	0.331	.200	0.00593098	0.243	.200	0.547
PCC - VMPFC	-0.06350145	0.262	.200	0.03295004	0.277	.200	0.219
HippocampL - VMPFC	-0.06210290	0.423	.200	-0.07471679	0.210	.200	0.711
HippocampR - VMPFC	-0.14549358	0.263	.200	-0.08592604	0.357	.200	0.592
MPFC - DMPFC	0.04758203	0.144	.200	0.05031548	0.217	.061	0.662
VMPFC - DMPFC	-0.14933994	0.206	.200	-0.12797460	0.315	.200	0.993
DMPFC - DMPFC	-0.29704619	0.266	.200	-0.35839600	0.287	.013*	0.219
IPLL - DMPFC	0.06456248	0.241	.200	-0.00709076	0.276	.052	0.136
Precuneus - DMPFC	0.00574835	0.200	.200	0.04051411	0.241	.200	0.504
PCC - DMPFC	-0.08453968	0.197	.200	0.02320282	0.278	.200	0.104
HippocampL - DMPFC	-0.18703326	0.367	.142	-0.13277264	0.422	.147	0.405
HippocampR - DMPFC	-0.22827116	0.440	.200	-0.21168364	0.468	.200	0.760
MPFC - IPLL	-0.02299235	0.219	.200	0.02937720	0.217	.034*	0.674
VMPFC - IPLL	-0.03706790	0.245	.200	-0.12641236	0.334	.116	0.473
DMPFC - IPLL	0.01507319	0.271	.200	0.01681740	0.356	.200	0.876
IPLL - IPLL	-0.13087277	0.344	.140	-0.16497260	0.337	.200	0.928
Precuneus - IPLL	0.01703594	0.353	.200	0.07197312	0.339	.187	0.473
PCC - IPLL	0.26026874	0.282	.200	0.05223032	0.352	.200	0.030*
HippocampR - IPLL	-0.19887987	0.381	.200	-0.23942680	0.463	.133	0.967
HippocampR - IPLL	-0.27910377	0.372	.144	-0.40910244	0.352	.200	0.335
MPFC - Precuneus	-0.03372316	0.199	.006*	0.04981690	0.166	.200	0.169

VMPFC - Precuneus	-0.03240632	0.173	.041*	-0.16148332	0.329	.200	0.091
DMPFC - Precuneus	-0.06816610	0.276	.200	-0.03468188	0.350	.200	0.773
IPLL - Precuneus	0.01643348	0.316	.015*	-0.01935044	0.368	.059	0.686
Precuneus - Precuneus	-0.07018358	0.287	.200	-0.13297036	0.336	.055	0.876
PCC - Precuneus	0.12501990	0.267	.170	0.02207460	0.231	.099	0.031*
HippocampL - Precuneus	-0.10881116	0.306	.200	-0.21495624	0.468	.200	0.570
HippocampR - Precuneus	-0.14579690	0.288	.200	-0.25213192	0.489	.200	0.662
MPFC - PCC	-0.04982197	0.251	.085	0.06534704	0.178	.178	0.045*
VMPFC - PCC	-0.06000003	0.222	.200	-0.06420230	0.270	.200	0.863
DMPFC - PCC	-0.10679003	0.318	.066	-0.14120096	0.273	.200	0.558
IPLL - PCC	0.11808003	0.351	.061	0.10075732	0.384	.001*	0.980
Precuneus - PCC	0.22890206	0.316	.200	0.22555496	0.417	.200	0.876
PCC - PCC	-0.17228045	0.270	.200	-0.05125964	0.310	.008*	0.076
HippocampL - PCC	-0.11947732	0.367	.194	-0.25854280	0.418	.136	0.119
HippocampR - PCC	-0.35405107	0.388	.200	-0.44107324	0.407	.138	0.673
MPFC - HippocampL	-0.01613671	0.199	.200	-0.02016908	0.131	.043*	0.748
VMPFC - HippocampL	0.09602058	0.220	.190	0.06537392	0.207	.144	0.674
DMPFC - HippocampL	-0.00147545	0.278	.200	-0.00398133	0.246	.015*	0.747
IPLL - HippocampL	0.10804568	0.315	.200	0.08238352	0.135	.019*	0.494
Precuneus - HippocampL	-0.00669494	0.214	.077	-0.00919944	0.227	.200	0.889
PCC - HippocampL	-0.01111039	0.277	.200	0.09427208	0.204	.200	0.185
HippocampL - HippocampL	-0.11763068	0.260	.017*	-0.04473412	0.320	.181	0.295
HippocampR - HippocampL	0.22041281	0.346	.200	0.08621208	0.309	.200	0.219
MPFC - HippocampR	-0.01582945	0.176	.200	0.00430216	0.191	.149	0.463
VMPFC - HippocampR	0.10306203	0.157	.200	0.05008740	0.221	.003*	0.473
DMPFC - HippocampR	0.03083210	0.236	.200	0.03700804	0.233	.200	0.993
IPLL - HippocampR	0.07766729	0.212	.200	0.06992796	0.186	.056	0.786
Precuneus - HippocampR	-0.04587845	0.271	.200	-0.01645120	0.148	.200	0.604
PCC - HippocampR	0.07143823	0.244	.200	0.08128080	0.237	.200	0.954
HippocampL - HippocampR	0.32710152	0.244	.157	0.19682600	0.220	.195	0.014*
HippocampR - HippocampR	-0.09222019	0.317	.200	-0.08106396	0.362	.200	0.941

Legend: MPFC – Medial prefrontal cortex; VMPFC - Ventral Medial prefrontal cortex; DMPFC - Dorsal Medial prefrontal cortex; IPLL – left inferior parietal lobe; PCC – Posterior cingulate cortex; HippocampL - Hippocampal formation Left; HippocampR - Hippocampal formation Right; ROIs – Regions of interest; SD – standard deviation; CID – Chronic insomnia disorder; HC – Healthy control; D – Kolmogorov-Smirnov test; U – Mann-Whitney test; *p<0.05.

One-sample Kolmogorov-Smirnov test results and between-group comparisons of Model 2

In model 2 which focused on the connectivity characteristics between the DMN and SN the coupling values of 11 connections, namely MPFC → Precuneus, Precuneus → PCC, HippocampL → PCC, HippocampR → PCC, AIR → PCC, dACCL → PCC, PCC → AIL, PCC → AIR, AIL → AIR, MPFC → dACCL, and the self-inhibitory connection of AIR statistically differed from zero in the HC group whereas in the patient groups the statistically significant connections were 5: HippocampL → PCC, HippocampL → HippocampR, AIR → AIL, MPFC → AIR, and AIL → AIR (Suppl. Table S5).

The two-sample Mann-Whitney showed that connectivity strengths of the Precuneus → PCC, HippocampR → HippocampL, dACCL → HippocampR, MPFC → AIR, and the self-inhibitory connection of HippocampL significantly differed between the HC and patient groups (Suppl. Table S5).

Supplementary Table S5: Group-wise and between-group connectivity strength analysis in chronic insomnia patients and healthy controls, according to Model 2.

**Connection**	**CID Group**			**HC Group**			**Between group comparison**
	**Mean**	**(±SD)**	**D**	**Mean**	**(±SD)**	**D**	**U**
MPFC - MPFC	-0.34418900	0.404	.100	-0.27239976	0.392	.186	0.434
Precuneus - MPFC	0.27783748	0.450	.200	0.26852296	0.507	.200	0.863
PCC - MPFC	0.08702462	0.451	.080	0.13241093	0.470	.200	0.378
HippocampL - MPFC	-0.12739668	0.642	.184	0.03631059	0.501	.200	0.352
HippocampR - MPFC	-0.24982900	0.397	.200	-0.22318332	0.447	.200	0.639
AIL - MPFC	-0.05670765	0.358	.200	-0.10574207	0.402	.200	0.650
AIR - MPFC	-0.11463699	0.362	.200	-0.06674112	0.380	.200	0.405
dACCL - MPFC	-0.02633016	0.407	.200	-0.03052380	0.489	.200	0.786
MPFC - Precuneus	0.03654699	0.258	.075	-0.06351512	0.426	.046*	0.773
Precuneus - Precuneus	-0.20811901	0.320	.110	-0.22169621	0.348	.200	0.850
PCC - Precuneus	-0.07542380	0.370	.200	0.00943604	0.317	.200	0.547
HippocampL - Precuneus	-0.11388477	0.426	.200	-0.06665436	0.384	.200	0.627
HippocampR - Precuneus	-0.31956694	0.405	.200	-0.36491336	0.481	.200	0.837
AIL - Precuneus	-0.20012319	0.236	.200	-0.14249400	0.384	.200	0.863
AIR - Precuneus	-0.13172900	0.266	.200	-0.12324884	0.332	.200	0.928
dACCL - Precuneus	0.06880691	0.309	.200	0.00529480	0.333	.200	0.378
MPFC - PCC	-0.01724990	0.194	.200	0.01460924	0.196	.200	0.415
Precuneus - PCC	0.30175151	0.302	.159	0.44116944	0.388	.033*	0.040*
PCC - PCC	-0.15487850	0.233	.200	-0.02236472	0.396	.085	0.066
HippocampL - PCC	0.02787265	0.351	.041*	-0.08328256	0.323	.016*	0.266
HippocampR - PCC	-0.30205584	0.288	.200	-0.22714436	0.358	.004*	0.159
AIL - PCC	-0.22627339	0.220	.200	-0.23766355	0.314	.200	0.824
AIR - PCC	-0.15171227	0.351	.200	-0.29498663	0.372	.021*	0.140
dACCL - PCC	-0.01488671	0.293	.134	-0.03684028	0.275	.002*	0.196
MPFC - HippocampL	0.00396418	0.156	.200	-0.00257284	0.125	.087	0.967
Precuneus - HippocampL	0.05620042	0.230	.200	-0.02269317	0.194	.121	0.213
PCC - HippocampL	0.01672467	0.167	.200	0.05759696	0.125	.200	0.327
HippocampL - HippocampL	-0.14281375	0.238	.060	0.02777060	0.371	.200	0.049*
HippocampR - HippocampL	0.28290226	0.346	.200	-0.01723542	0.306	.200	0.001*
AIL - HippocampL	-0.10983368	0.280	.197	-0.07583449	0.172	.200	0.837
AIR - HippocampL	0.06773877	0.254	.200	-0.02303337	0.263	.200	0.280
dACCL - HippocampL	0.01355258	0.248	.080	0.06838348	0.219	.200	0.201
MPFC - HippocampR	0.02433279	0.139	.200	-0.02094514	0.152	.115	0.201

Precuneus - HippocampR	0.03334123	0.244	.200	0.03637070	0.180	.118	0.980
PCC - HippocampR	0.09293841	0.179	.054	0.09135107	0.246	.084	0.811
HippocampL - HippocampR	0.23759258	0.275	.042*	0.22270369	0.287	.200	0.954
HippocampR - HippocampR	-0.08238985	0.286	.200	0.06827832	0.371	.083	0.164
AIL - HippocampR	-0.01849390	0.321	.200	-0.03661952	0.209	.200	0.863
AIR - HippocampR	0.10111949	0.232	.200	0.01186686	0.185	.190	0.076
dACCL - HippocampR	-0.04720404	0.198	.200	0.12765807	0.180	.200	0.003*
MPFC - AIL	0.00472087	0.151	.170	-0.03408876	0.185	.200	0.558
Precuneus - AIL	-0.06100968	0.210	.200	-0.02972831	0.268	.200	0.650
PCC - AIL	0.11309668	0.225	.143	0.07643758	0.233	.021*	0.547
HippocampusL - AIL	-0.08089990	0.314	.200	0.01298256	0.232	.200	0.164
HippocampusR - AIL	-0.13033390	0.280	.200	-0.10705652	0.264	.200	0.954
AIL - AIL	-0.10333555	0.245	.071	-0.10767696	0.257	.200	0.798
AIR - AIL	0.11293852	0.406	.001*	0.09011228	0.489	.200	0.902
dACCL - AIL	0.09524708	0.346	.200	0.10229108	0.385	.094	0.902
MPFC - AIR	0.06669617	0.172	.019*	-0.06238074	0.221	.200	0.014*
Precuneus - AIR	-0.06504395	0.213	.200	0.01813836	0.234	.200	0.164
PCC - AIR	-0.01701311	0.269	.200	0.14838718	0.242	.001*	0.039
HippocampL - AIR	-0.06923003	0.348	.200	-0.02136693	0.285	.200	0.537
HippocampR - AIR	-0.05372997	0.306	.200	-0.02670076	0.308	.200	0.570
AIL - AIR	0.29433685	0.363	.049*	0.39894892	0.266	.017*	0.213
AIR - AIR	-0.06039425	0.283	.200	-0.08962520	0.331	.015*	0.902
dACCL - AIR	0.04741255	0.284	.200	0.00226092	0.345	.200	0.798
MPFC - dACCL	-0.01012969	0.173	.053	-0.04856971	0.251	.006*	0.558
Precuneus - dACCL	0.03458477	0.303	.200	0.00796564	0.266	.200	0.686
PCC - dACC:	0.00883743	0.229	.200	0.06917600	0.272	.200	0.387
HippocampL - dACCL	-0.00947939	0.392	.200	-0.06149656	0.347	.200	0.824
HippocampR - dACCL	-0.14842501	0.260	.200	-0.21181956	0.296	.200	0.526
AIL - dACCL	0.21930661	0.340	.200	0.23350552	0.341	.200	0.889
AIR - dACCL	0.11396158	0.395	.200	0.03181144	0.360	.200	0.303
dACCL - dACCL	-0.13037039	0.275	.116	-0.13105448	0.265	.143	0.850

Legend: MPFC – Medial prefrontal cortex; PCC – Posterior cingulate cortex; HippocampL - Hippocampal formation Left; HippocampR - Hippocampal formation Right; AIL - Anterior insula Left; AIR - Anterior insula Right; dACC L – Left dorsal anterior cingulate cortex; ROIs – Regions of interest; SD – standard deviation; CID – Chronic insomnia disorder; HC – Healthy control; D – Kolmogorov-Smirnov test; U – Mann-Whitney test; *p<0.05.

One-sample Kolmogorov-Smirnov test results and between-group comparisons of Model 3

The final model exploring the connectivity patterns among the DMN, SN and ECN showed that in the HC group there were 4 connections which statistically differed from zero: MPFC → PCC, PCC → HippocampL, Cingulate Gyrus → DLPFC, PCC → Cingulate Gyrus while in the patient group the self-inhibition of the MPFC, DLPFC → HippocampR, and MPFC → DLPFC connections were statistically significant against zero (Suppl. Table S6).

In addition, the between-group comparison determined statistically significant differences in the HippocampL → PCC and DLPFC → HippocampR connections between the HC and CID groups (Suppl. Table S6).

Supplementary Table S6: Group-wise and between-group connectivity strength analysis in chronic insomnia patients and healthy controls, according to Model 3.

**Connection**	**CID Group**			**HC Group**			**Between group comparison**
	**Mean**	**(±SD)**	**D**	**Mean**	**(±SD)**	**D**	**U**
MPFC - MPFC	-0.18705830	0.366	.045*	-0.12705568	0.315	.200	0.686
PCC - MPFC	0.39555616	0.404	.200	0.50722080	0.293	.200	0.127
HippocampL - MPFC	-0.09569303	0.587	.079	-0.19053060	0.656	.200	0.786
HippocampR - MPFC	-0.35027368	0.508	.200	-0.19152052	0.531	.135	0.443
DLPFC - MPFC	-0.13977445	0.272	.156	-0.18509360	0.601	.098	0.615
Cingulate Gyrus - MPFC	0.10669489	0.367	.200	0.13828684	0.474	.200	0.723
MPFC - PCC	-0.03711872	0.233	.200	0.03780490	0.261	.002*	0.735
PCC - PCC	-0.283635097	0.300	.200	-0.319495520	0.336	.200	0.863
HippocampL - PCC	0.02042598	0.410	.200	-0.23123034	0.625	.200	0.040*
HippocampR - PCC	-0.36819158	0.431	.200	-0.42350068	0.726	.200	0.434
DLPFC - PCC	0.06306060	0.364	.200	-0.17889064	0.557	.200	0.119
Cingulate Gyrus - PCC	0.00859629	0.339	.200	0.02450484	0.396	.200	0.723
MPFC - HippocampL	0.01033425	0.159	.132	-0.00822012	0.155	.200	0.415
PCC - HippocampL	0.04118383	0.238	.200	0.13637188	0.199	.003*	0.154
HippocampL - HippocampL	0.00907439	0.351	.200	-0.02359892	0.485	.200	0.954
HippocampR - HippocampL	0.17331281	0.368	.200	0.16744736	0.295	.200	0.735
DLPFC - HippocampL	-0.05584371	0.276	.200	0.07161511	0.215	.200	0.094
Cingulate Gyrus - HippocampL	0.00455832	0.301	.200	-0.05154971	0.219	.200	0.280
MPFC - HippocampR	0.01945081	0.151	.200	0.03965496	0.161	.200	0.674
PCC - HippocampR	0.11526381	0.175	.200	0.04318564	0.207	.200	0.245
HippocampL - HippocampR	0.17931430	0.288	.200	0.11830332	0.296	.200	0.504
HippocampR - HippocampR	0.07008058	0.435	.200	-0.10363324	0.436	.200	0.169
DLPFC - HippocampR	-0.11001877	0.258	.035*	0.18369974	0.204	.195	<0.001**
Cingulate Gyrus - HippocampR	0.04808966	0.270	.200	-0.00508110	0.217	.200	0.537
MPFC - DLPFC	-0.02498813	0.227	.009*	-0.01414012	0.111	.194	0.954
PCC - DLPFC	0.07226248	0.272	.113	0.18270000	0.216	.065	0.213
HippocampL - DLPFC	-0.06770071	0.473	.185	-0.07769098	0.386	.200	0.889
HippocampR - DLPFC	-0.10624003	0.416	.200	-0.21396323	0.412	.200	0.201
DLPFC - DLPFC	-0.07738568	0.279	.134	-0.01960608	0.356	.200	0.711
Cingulate Gyrus - DLPFC	0.05162280	0.323	.092	0.10093998	0.382	.005*	0.091
MPFC - Cingulate Gyrus	-0.03346160	0.192	.200	0.04699630	0.174	.200	0.098
PCC - Cingulate Gyrus	0.05646681	0.348	.200	-0.00006612	0.326	.001*	0.760

HippocampL - Cingulate Gyrus	0.02593358	0.483	.200	0.08565003	0.428	.200	0.674
HippocampR - Cingulate Gyrus	-0.17750184	0.464	.200	-0.31683416	0.506	.200	0.335
DLPFC - Cingulate Gyrus	-0.02599710	0.291	.200	0.02083308	0.319	.200	0.581
Cingulate Gyrus - Cingulate Gyrus	-0.27865774	0.333	.200	-0.17819112	0.279	.200	0.360

Legend: MPFC – Medial prefrontal cortex; PCC – Posterior cingulate cortex; HippocampL - Hippocampal formation Left; HippocampR - Hippocampal formation Right; DLPFC – Dorsolateral prefrontal cortex; ROIs – Regions of interest; SD – standard deviation; CID – Chronic insomnia disorder; HC – Healthy control; D – Kolmogorov-Smirnov test; U – Mann-Whitney test; *p<0.05; **p<0.001.
